# Examining the immune response of participants receiving Modified Vaccinia Ankara vaccine in Africa (MpoxVax AFRIVAC): a prospective cohort study – protocol

**DOI:** 10.1136/bmjopen-2026-120064

**Published:** 2026-07-31

**Authors:** Joanne Byrne, Winters Muttamba, Alain Balola Ntaboba, Bariki Mtafya, Mudarshiru Bbuye, Arthur Kalyebi Watelo, Elena Alvarez Barco, Ayleen Mufudza, Barnabas Bakamutumaho, Eoin R Feeney, Andrew Ekii Obuku, Nyanda Elias Ntinginya, Patrick D M C Katoto, Wilber Sabiiti, Bruce Kirenga, Patrick W G Mallon

**Affiliations:** 1 Centre for Experimental Pathogen Host Research (CEPHR), University College Dublin, Dublin, Ireland; 2 Department of Infectious Diseases, St Vincent’s University Hospital, Dublin, Ireland; 3 Interdisciplinary Consortium for Epidemics Research (ICER), Kampala, Uganda; 4 Makerere University Lung Institute, Kampala, Central Region, Uganda; 5 Center for Tropical Diseases and Global Health, Faculty of Medicine, Catholic University of Bukavu, Bukavu, South-Kivu, Congo (the Democratic Republic of the); 6 National Institute for Medical Research (NIMR)-Mbeya Medical Research Center, Mbeya, Tanzania, United Republic of; 7 Medical Research Council/Uganda Virus Research Institute and London School of Hygiene, Tropical Medicine Uganda Research Unit, Entebbe, Uganda; 8 Uganda National Health Research Organization, Berkeley Lane, Entebbe, Uganda; 9 London School of Hygiene and Tropical Medicine, London, UK; 10 Division of Epidemiology and Biostatistics, Department of Global Health, Faculty of Medicine and Health Sciences, Stellenbosch University, Stellenbosch, Western Cape, South Africa; 11 Cochrane South Africa, South African Medical Research Council, Cape Town, Western Cape, South Africa; 12 Division of Infection and Global Health, School of Medicine, University of St Andrews, St Andrews, UK; 13 Department of Medicine, Makerere University College of Health Sciences, Kampala, Central Region, Uganda

**Keywords:** INFECTIOUS DISEASES, VIROLOGY, Vaccination

## Abstract

**Introduction:**

Mpox remains an ongoing global health concern, driven by an ongoing clade I outbreak in Central Africa, which has caused substantial morbidity and mortality. Despite deployment of the Modified Vaccinia Ankara-Bavarian Nordic (MVA-BN) vaccine, data on the durability of vaccine-induced immune responses remain limited, particularly in African populations, women and people with HIV, highlighting the need for context-specific immunogenicity data.

**Methods and analysis:**

Mpox-Vax AFRIVAC is a prospective, multicentre observational cohort study conducted in Uganda, Tanzania and the Democratic Republic of the Congo (DRC). Adults eligible for MVA-BN vaccination for mpox prevention or who received a first dose within 28 days prior to enrolment are followed for 48 weeks. Serial blood samples are collected to quantify binding antibody responses and assess their durability over time. The primary outcomes are mean geometric MVA-BN-specific antibody titres at week 48 and the rate of antibody decay from week 6 to week 48. Secondary outcomes include antibody kinetics at intermediate time points, factors associated with immune responses, breakthrough infection and vaccine safety. Analyses will use descriptive and longitudinal statistical methods to evaluate immune response trajectories and associated host factors, including HIV status, age and sex.

**Ethics and dissemination:**

The study is conducted in accordance with the Declaration of Helsinki, registered at the Pan African Clinical Trials Registry (PACTR202601855406764) and publications will be in accordance with the recommendations of the International Committee of Medical Journal Editors. The study has received ethical approvals in Uganda, DRC and Tanzania. Approval for Uganda has been obtained from Uganda Virus Research Institute Research and Ethics Committee (GC/127/1062) and Uganda National Council for Science and Technology (HS5575ES), for DRC from the Institutional Committee of Health ethics (UCB/CIES/PB/001/2025) and for Tanzania from the National Institute for Medical Research (NIMR/HQ/R.8a/Vol.IX/5187).

**Trial registration number:**

PACTR202601855406764.

**STRENGTHS AND LIMITATIONS OF THIS STUDY**

- This is a prospective multicentre cohort study conducted across African countries directly affected by the ongoing clade Ib mpox outbreak.
- Predefined recruitment quotas are used to ensure representation of women and people with HIV.
- Serial longitudinal sampling over 48 weeks enables assessment of the durability of Modified Vaccinia Ankara-Bavarian Nordic-induced antibody responses using harmonised methods across African and European cohorts.
- Standardised serological testing is performed using a validated quantitative electrochemiluminescence immunoassay.
- As an observational study embedded within standard-of-care vaccination programmes, causal inference regarding vaccine effectiveness is limited.

## Introduction

 Mpox infections, caused by the Monkeypox virus (MPXV), remain a major and evolving global public health challenge. In May 2022, an unprecedented outbreak marked the first sustained autochthonous transmission of clade IIb MPXV in non-endemic regions, prompting the WHO to declare mpox a Public Health Emergency of International Concern (PHEIC) in July 2022.^1^ To date, over 100 000 cases of clade IIb mpox have been reported, with transmission occurring predominantly through sexual contact among gay and bisexual men who have sex with men (gbMSM).^2^ In parallel, an outbreak of clade Ib MPXV in Central Africa has intensified and has resulted in almost 65 000 cases and a reported case fatality ratio of 2.3% in endemic regions.^3^ In contrast to the 2022 outbreak, women have accounted for more than half of those affected.^4^ This led the WHO to declare a second PHEIC in August 2024, establishing the African outbreak as a dominant global public health priority.^5^

In the context of the PHEIC a third-generation non-replicating smallpox vaccine, Modified Vaccinia Ankara-Bavarian Nordic (MVA-BN), received emergency use authorisation for mpox pre-exposure and postexposure prophylaxis. Short-term immunogenicity data from phase I–III clinical trials demonstrated robust antibody responses,^6–8^ and observational studies from the clade IIb outbreak have estimated that two doses confer substantial protection against mpox infection.^9^ However, these data are largely limited to early postvaccination time points and predominantly reflect populations affected during the 2022 outbreak, namely Caucasian gbMSM in high-income, non-endemic settings. As a result, their generalisability to African populations affected by clade Ib MPXV remains uncertain.

Emerging evidence indicates that circulating and neutralising antibody responses induced by MVA-BN vaccination may wane substantially within 2 years postvaccination, raising concerns about durability of vaccine-induced protection.^10–13^ Consistent with this, increasing reports of breakthrough mpox infections in vaccinated individuals further underscore uncertainty surrounding the longevity of protective immunity.^14 15^ These concerns take on particular significance in settings where host factors may accelerate immune decay, notably in regions with a high prevalence of HIV such as Uganda and the Democratic Republic of the Congo (DRC).^16 17^ Several studies have demonstrated that people with HIV (PWH) exhibit lower peak binding and neutralising antibody titres following MVA-BN vaccination, more rapid antibody decay and associations between reduced CD4+ T cell counts and impaired humoral responses.^10 18–20^ This is especially concerning given that PWH experience a disproportionate burden of severe mpox disease and mortality.^21^ Despite this, data on vaccine immunogenicity in African populations, women and individuals with immunosuppression remain extremely limited.

Understanding the durability of immune responses following mpox vaccination in the populations most affected by ongoing transmission is essential to inform public health policy. Such data are required to optimise vaccination schedules, determine the need for booster doses and identify subgroups at increased risk of waning immunity. Without robust, context-specific immunogenicity data, vaccination strategies risk being extrapolated from populations with fundamentally different epidemiological and immunological profiles.

The MpoxVax AFRIVAC study is a prespecified African extension of the MpoxVax phase IV clinical trial in Ireland (EUCT number: 2023–5 07 881-19-00), implemented using harmonised protocols while addressing region-specific epidemiological and immunological questions in areas affected by the ongoing clade I mpox outbreak. Leveraging established clinical research infrastructure across multiple African sites, this prospective cohort study aims to evaluate the durability of MVA-BN specific antibody responses up to 1 year following vaccination and to identify host and clinical factors associated with immune response kinetics in an African population at risk of mpox.

## Objectives

### Primary objective

1. To assess the rate of change (durability) of, and with, Orthopoxvirus (OPXV)-specific antibodies at week 48 post first vaccination with MVA-BN.

### Secondary objectives

To determine the:

1. The baseline seroprevalence of OPXV-specific antibodies consistent with prior mpox infection at week 0.
2. The development of OPXV-specific binding antibody responses at week 4 and week 6 following MVA-BN vaccination.
3. To assess the durability of OPXV-specific antibodies at week 24, measured as geometric mean antibody titres and fold-change from week 6.
4. Demographic and clinical factors associated with OPXV-specific antibody responses and durability at week 48 postvaccination with MVA-BN.
5. To assess the rate of change (durability) of OPXV-specific antibodies at week 48 post first vaccination with MVA-BN in understudied populations, including women and people with HIV.
6. The prevalence of asymptomatic seroconversion consistent with incident mpox infection by week 48 postvaccination.
7. To describe the frequency and severity of adverse events following MVA-BN vaccination through week 48.

## Methods and analysis

### Study design

This is a prospective, multicentre observational cohort study evaluating immune responses following administration of the MVA-BN vaccine for prevention of mpox infection. Participants will be enrolled either prior to receipt of the first MVA-BN dose or within 28 days following receipt of the first dose administered as part of standard-of-care vaccination programmes. The study was designed as a prespecified African extension of the MpoxVax phase IV clinical trial currently underway in Ireland to characterise the magnitude and durability of vaccine-induced immune responses over a 48-week follow-up period and to identify host factors associated with immunogenicity in an African setting.

### Study setting

Participants will be recruited from mpox vaccination centres established by ministries of health in mpox-affected regions in three African countries: Uganda, Tanzania and the DRC. The mpox vaccination centres have been established by ministries of health based on the prevailing epidemiological data in the study countries and thus are mpox hotspots where there is an increased risk of mpox transmission. The vaccination exercise by the ministries of health is targeting high risk individuals such as PWH, sex workers among others. Clinical assessments and follow-up will be conducted at Makerere University Lung Institute (MLI) in Uganda, the National Institute for Medical Research (NIMR) in Tanzania and the Catholic University of Bukavu in the DRC. Laboratory processing will be done at local laboratories, and centralised immunological analyses will be conducted at the Uganda Virus Research Centre in Uganda and the Centre for Experimental Pathogen Host Research, University College Dublin.

### Participant recruitment and enrolment

Participants will be recruited at the ministry of health vaccination centres established in mpox hotspots identified through national surveillance. The study teams will work closely with the vaccination teams and community mobilisers to boost recruitment. Eligibility of the participants will be verified through vaccination cards, registration forms and national electronic vaccination records where available. Recruitment quotas have been predefined to ensure adequate representation of women and PWH, who are among the most affected in the ongoing clade 1b mpox outbreak. The study population has an a priori quota of:

- 25% participants who are people with HIV.
- 50% female participants.

Written informed consent will be obtained from all participants by trained study staff prior to enrolment using participant information materials provided in appropriate local languages, with verbal explanation to ensure understanding. Consent will be documented in accordance with regulatory requirements.

### Eligibility criteria

#### Inclusion criteria

In order to be eligible for participation in the study, the individuals must be:

1. Adults aged 18 years or older.
2. Eligible for MVA-BN vaccination for mpox prevention as per the national guidelines or have received the first dose of MVA-BN within 28 days prior to enrolment.
3. Able to understand the study procedures, comply with the study procedures and voluntarily agree to participate by giving written informed consent for the trial.

#### Exclusion criteria

Individuals will be excluded if they:

1. Are unable or unwilling to provide informed consent.
2. Have contraindications to MVA-BN vaccination.
3. Have known hypersensitivity to vaccine components.
4. Are pregnant or breastfeeding at screening.
5. Have clinical signs or prior laboratory-confirmed diagnosis of mpox.
6. Are participating in another interventional vaccine or therapeutic study.
  These exclusions will be aligned with national vaccination guidance and existing safety data.

### Study medication

The MVA-BN vaccine will be administered by the ministry of health vaccination teams as part of standard-of-care for mpox response. As this will be given outside of study activities, this study is considered an observational study. National Ministry guidelines recommended one dose of 0.5 mL of MVA-BN administered subcutaneously, with consideration of a second dose approximately 4 weeks later if sufficient vaccine stocks became available. However, due to vaccine supply constraints during the study period, all participants in this cohort received a single dose of MVA-BN only. No study-mandated modifications to vaccination schedules will be introduced. The vaccine brand, batch number, administration route, site and date will be recorded for all participants.

### Study procedures

Participants will attend up to five scheduled study visits over a 48-week follow-up period, with the number and timing of visits determined by vaccination status at enrolment. The scheduled visits will include baseline, early postvaccination, intermediate and long-term follow-up assessments. At each visit, clinical data will be collected, including mpox exposure history, symptoms, medical history, concomitant medications and adverse events. HIV-related clinical variables, including antiretroviral therapy status and history of advanced HIV disease (defined as CD4+ T cell count <200 cells/mm³), will be collected for all participants living with HIV. Blood samples will be obtained at all scheduled visits for immunological analyses. Unscheduled visits are permitted at any time for the assessment of suspected mpox infection or adverse events.

**Figure 1:**
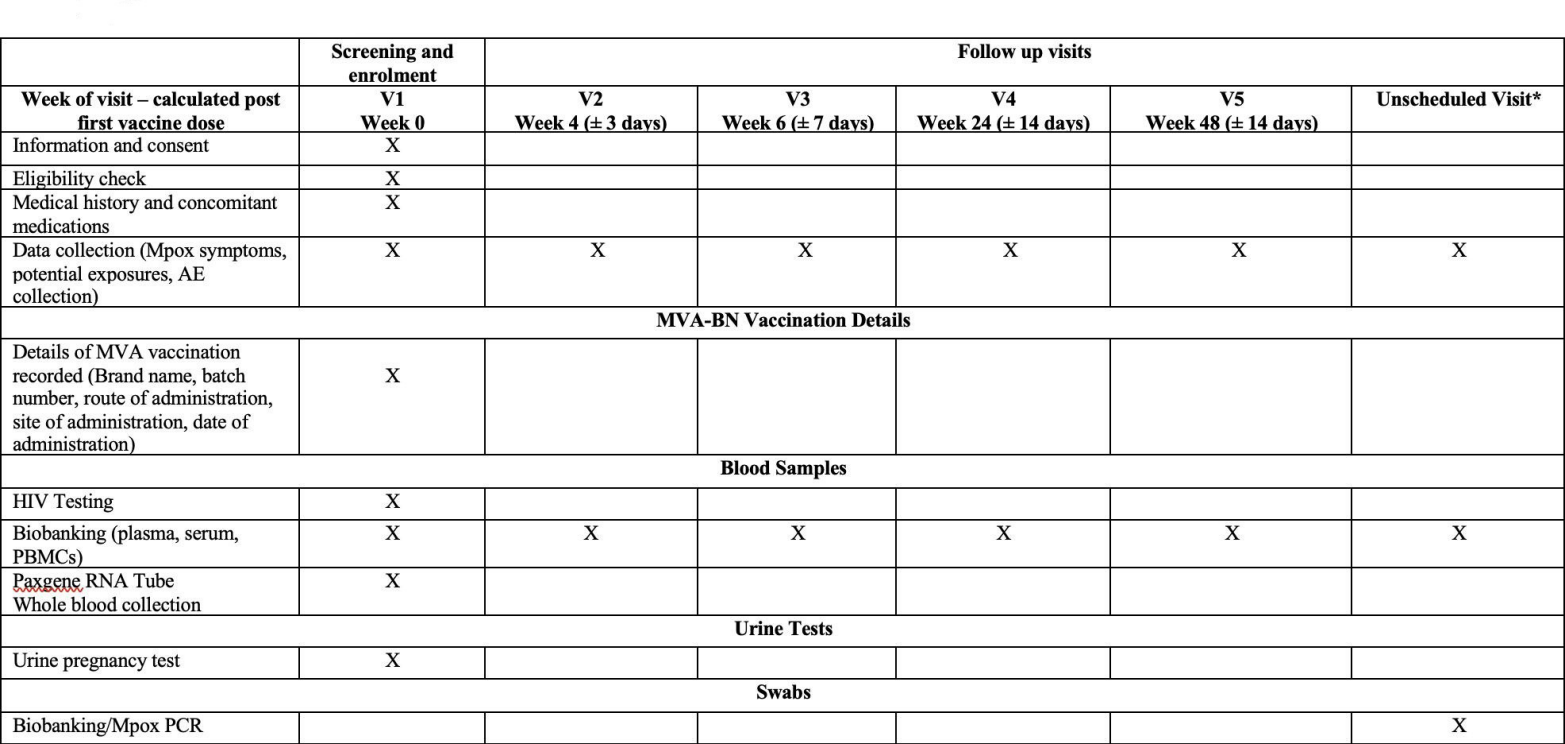
Schedule of visits. Subjects will attend a total of five clinical trial visits over 48 weeks of study as outlined in the schedule of visits above. Visit 2 should be scheduled within a window period of ±3 days, visit 3 within a window period of ±7 days, while visit 4 and visit 5 should be scheduled within a window period of ±2 weeks. Week 0 will align as closely as possible to the date of the first vaccination dose. Week of visit is calculated post date of first MVA dose. *Unscheduled visit to facilitate diagnosis and evaluation of mpox (vaccine failure cases) or evaluation of adverse events. Unscheduled visits will include clinically indicated laboratory testing and if mpox is suspected, biobanking of plasma, serum and PBMCs and swab of mpox lesions for future related work. AE, adverse event; MVA-BN, Modified Vaccinia Ankara-Bavarian Nordic; PBMC, peripheral blood mononuclear cell.

For participants enrolled prior to receipt of the first MVA-BN dose, the baseline (week 0) visit will be aligned as closely as possible to the date of first vaccination, followed by visits at weeks 4, 6, 24 and 48 postvaccination. A detailed schedule of visits and procedures for these participants is shown in figure 1.

Participants who will have received their first MVA-BN dose within 28 days prior to enrolment will not undergo a week 0 visit. In these participants, the week 4 visit will constitute the first study visit, followed by visits at weeks 6, 24 and 48 postvaccination, as detailed in the online supplemental appendix.

Participant retention strategies include engagement through community mobilisers and local study teams, flexible visit scheduling within predefined visit windows (figure 1) and ongoing communication with participants throughout follow-up. Risk Communication and Community Engagement (RCCE) teams and Community Advisory Boards (CABs) at participating sites will also support participant engagement and retention during the study period. These activities were designed to support participant trust, address vaccine-related misconceptions and facilitate long-term retention during the 48-week follow-up period.

### Study timeline

Recruitment commenced in Uganda and the DRC in January 2025 following completion of regulatory approvals and site initiation activities. Although regulatory approvals were obtained in all participating countries, participant recruitment in Tanzania did not proceed due to limited vaccine availability. Enrolment is complete, with final participant follow-up anticipated to be completed in June 2026.

### Safety monitoring

Adverse events will be collected at all study visits through participant self-reporting and clinical assessment. Serious adverse events and suspected unexpected serious adverse reactions will be reported to sponsors and regulatory authorities in accordance with Good Clinical Practice (GCP) and national regulations.

### Laboratory procedures

Blood samples will be collected at each visit for serological and cellular immune analyses, including plasma, serum and peripheral blood mononuclear cells (PBMCs). Lesion swabs will be collected when clinically indicated for mpox PCR testing and biobanking. Samples will be processed locally and stored in monitored biobanking facilities prior to shipment for central analysis at Uganda Virus Research Institute.

Antibody responses will be quantified using a validated quantitative immunoassay developed at the Centre for Experimental Pathogen Host Research. This electrochemiluminescence assay measures IgG titres to three OPXV antigens (MPXV B6R, MPXV A27L and VACV B5) and has been previously validated for the quantification and differentiation of antibody responses following mpox infection and MVA-BN vaccination.^12^

Functional neutralisation assays, gene expression analyses and additional cellular immunological analyses may be performed on selected subsets of stored samples in future studies. PBMC samples are being prospectively collected to support exploratory cellular immunological analyses in selected participant subsets and for future ethically approved ancillary studies related to mpox immunology and vaccine responses. Sample subsets will be selected to ensure representation across key clinical and demographic groups and longitudinal study timepoints.

### Endpoints

#### Primary endpoint

1. Mean geometric OPXV-specific antibody titres at week 48 post vaccination with MVA-BN in a population recruited in Africa.
2. Mean rate of change in OPXV-specific antibody titres post week 6 to week 48 in a population recruited in Africa.

#### Secondary endpoints

1. Proportion of participants with detectable OPXV-specific binding IgG at week 0 consistent with prior mpox infection.
2. Mean geometric OPXV-specific antibody titres at week 4 and week 6 post MVA-BN vaccination
3. Mean geometric OPXV-specific IgG titres at week 24 and fold-change from week 6.
4. Association between predefined demographic and clinical variables and OPXV-specific antibody titres at week 48.
5. Mean geometric OPXV-specific antibody titres at week 48 post vaccination with MVA-BN in women and in PWH and mean rate of change in OPXV-specific antibody titres post week 6 to week 48 in women and in PWH.
6. Proportion of participants with serological evidence of incident mpox infection between week 0 and week 48.
7. Proportion of participants reporting any adverse event and severity within each follow-up interval (0–4, 4–6, 6–24, 24–48 weeks).

### Sample size

We aim to have a 95% chance of obtaining a 90% CI (ie, an overall probability of achieving the desired precision and capturing the true mean with power of 0.95×0.9) whose half-width is at most 50 GMT. Assuming an expected SD of 485 GMT at week 48 (based on simulations using data from the Pittman *et al* phase III efficacy trial of MVA-BN vaccine),^8^ a sample size of approximately 300 participants would be required to achieve the desired level of accuracy around the mean GMT at week 48. To account for a potential 9% dropout rate, 330 participants will be recruited across three clinical sites.

### Statistical analysis

#### General principles

All analyses will be conducted using the full analysis set. Descriptive and exploratory analyses, including summary statistics, plots and data visualisation, will be performed using R (V.4.5.2 or higher). Multiple imputation for missing primary endpoint data and any associated confirmatory analyses will be performed using SAS (V.9.4 or higher) to ensure compliance with standard regulatory practices. Descriptive summary statistics will include the number of subjects (n), mean, SD, median, first and third quartiles (Q1 and Q3), minimum and maximum for continuous variables. Categorical variables will be summarised using counts and percentages, calculated either from the total number of subjects in the analysis population or from the number of subjects with available assessments, as appropriate. Two-sided 95% CIs will be presented where specified. All analyses will be performed in accordance with the study objectives and endpoints. Sensitivity analyses using multiple imputation will be performed in SAS for the primary endpoint. No sensitivity analyses are planned for secondary endpoints.

### Primary endpoint analysis

The primary objective of the study is to assess the durability of, and factors associated with, MVA-BN-specific antibodies at week 48 following a single vaccination dose. The primary endpoints of this study are

- Mean geometric OPXV-specific antibody titres at week 48 postvaccination with MVA-BN in a population recruited in Africa.
- Mean rate of change in OPXV-specific antibody titres from week 6 to week 48 in a population recruited in Africa.

Antibody titres measured from baseline through Week 48 will be analysed using a linear mixed-effects model with log-transformed antibody titres as the dependent variable. To reflect the expected biological pattern of antibody titres following vaccination, time will be modelled as a continuous variable with a prespecified change point at week 6. This specification allows separate linear components for the expansion phase (baseline to week 6) and the waning phase (week 6 to week 48). Week 6 was selected a priori as the change point as this time point is expected to approximate peak vaccine-induced antibody responses following MVA-BN vaccination, thereby separating the early antibody expansion phase from the subsequent waning phase. The model will include fixed effects for time in each interval and a random intercept to account for within-participant correlation arising from repeated measurements.

The slope corresponding to the week 6 to week 48 interval will represent the primary durability parameter. An unstructured covariance matrix will be used to model the correlation among repeated measures within participants. If the unstructured covariance model fails to converge, alternative covariance structures, such as Toeplitz or heterogeneous first-order autoregressive (AR(1)), will be considered. Selection among competing covariance structures will be guided by the Akaike information criterion, balancing model fit and parsimony. Denominator df for fixed effects will be estimated using the Kenward-Roger approximation. The fitted mixed-effects model will be used to derive the model-estimated geometric mean titre at week 48 by back-transforming the least squares mean from the log scale, together with corresponding SEs and 95% CIs. The fixed-effect slope for the week 6 to week 48 interval will provide the estimated mean rate of change in log-transformed antibody titres during the waning phase, with corresponding 95% CIs. Results will be presented as model-estimated geometric mean titres with 95% CIs, estimated week 6 to week 48 slopes with 95% CIs and graphical displays of observed individual data with superimposed model-fitted trajectories over time.

The primary analysis of the endpoints will be conducted using observed data only (ie, without imputation of missing values) within the likelihood-based mixed-effects modelling framework. This approach accommodates incomplete repeated-measures data under the missing-at-random (MAR) assumption and provides the primary estimates of the geometric mean MVA-BN antibody titre at week 48 and the rate of change from week 6 to week 48. To inform the sensitivity analyses using multiple imputation, the extent and pattern of missing data will be summarised, including the proportion of participants with missing antibody titre measurements at each study visit and, where available, documented reasons for missingness. As part of the sensitivity analysis, multiple imputation will be performed under the assumption of MAR. Missing log-transformed antibody titres will be imputed using a model-based approach that includes all variables specified in the primary analysis model and incorporates the longitudinal structure of the repeated measurements. The number of imputations will depend on the overall proportion of missing data: 10 imputations will be generated if missingness is less than 10%, and 20 imputations will be generated if missingness is 10% or greater. Each imputed dataset will be analysed using the same piecewise mixed-effects model specified for the primary analysis. Estimates and associated standard errors will be combined across imputations using Rubin’s rules to obtain overall estimates and 95% CIs. Consistency between the primary observed-data analysis and the multiple imputation analysis will be assessed by comparing point estimates and corresponding 95% CIs for the week 48 geometric mean titre and the rate of change from week 6 to week 48. Differences will be considered material if they result in a meaningful change in the magnitude, direction or interpretation of the study conclusions.

### Secondary endpoints

The proportion of participants with detectable OPXV-specific binding IgG at week 0 consistent with prior mpox infection is a predefined secondary endpoint. Baseline seropositivity will be defined using MPXV B6R antibody responses according to previously derived serological thresholds from the validated electrochemiluminescence immunoassay.^12^ As these thresholds were originally derived in non-African populations, their performance and distribution within the African cohort will be evaluated and threshold refinement may be considered if required based on observed assay performance characteristics in this population. Baseline serostatus may additionally be incorporated as an exploratory covariate in analyses evaluating factors associated with vaccine-induced immune responses.

HIV-related clinical variables, including history of advanced HIV disease (defined as HIV infection with CD4+ T cell count <200 cells/mm³) may be evaluated as exploratory covariates in analyses assessing factors associated with vaccine-induced immune responses. These analyses relate to the predefined secondary endpoints assessing demographic and clinical factors associated with immunogenicity. Additional secondary endpoint analyses are described in detail in the statistical analysis plan.

### Data management

Data will be collected, generated and processed following the Findable, Accessible, Interoperable, Reusable (FAIR) principles according to a data management plan that will be established at the start of the project. The data will be managed within and outside the consortium according to the data management plan, describing the following steps: (1) description of data, (2) metadata standard use for the data, (3) policies for access, sharing and privacy, (4) policies for reuse, redistribution, derivative and (5) plans for archiving and preservation. Briefly, electronic versions of the hard copy data collection tools will be developed and deployed on password protected computers to support electronic data collection. Data will be uploaded on a secure server based at MLI, where a data manager will be at regular intervals do data quality checks for accuracy, completeness and consistency. The data manager will generate any data quality queries and feedback to the data collection teams to resolve the raised queries.

### Patient and public involvement

Patients and members of the public were involved at an early stage in the development of the parent Mpox-Vax Ireland study, contributing to the design of the study protocol and participant information materials. This involvement informed the research questions, outcome measures and recruitment strategy, with specific focus on optimising the Participant Information Leaflet to ensure clarity, accessibility and acceptability for the target population.

MpoxVax AFRIVAC is a prespecified African extension of the Irish study using a harmonised protocol, and the study will therefore not be redesigned with new local patient and public involvement during the protocol development phase. However, a multidisciplinary team will be established at each participating African site before the implementation of the study, referred to the RCCE team. This team will be actively involved in participant engagement, recruitment and retention throughout the study, providing ongoing feedback on study procedures. The RCCE team will include the community representatives such as the community leaders, community mobilisers, community health workers as well as the local stakeholders (facilitators) including the local health authorities, healthcare facility representatives, selected institutional staff members from MLI, Université Catholique de Bukavu (UCB) and NIMR) as well as the study personnel. Furthermore, a CAB will be established at each of the study sites to act as liaison between the study and the community. In close collaboration with the RCCE, the CAB board will serve as a learning platform where scientists and community representatives exchange approaches to facilitate dialogue with participants, address misconceptions about mpox, counter rumours on vaccination and sustain community trust.

Study findings will be disseminated through peer-reviewed publications and scientific presentations, and site teams will support communication of key results to participants and relevant local communities in an appropriate and accessible format. The dissemination activities will be supported by the CAB.

### Ethics and dissemination

MpoxVax AFRIVAC is performed in accordance with the Declaration of Helsinki and according to GCP. The study was registered at the Pan African Clinical Trials Registry (PACTR202601855406764) on 5th January 2026 and has ethical approval at three clinical sites.

The study has received ethical approvals in Uganda, DRC and Tanzania. Approval for Uganda has been obtained from Uganda Virus Research Institute Research and Ethics Committee (GC/127/1062) and Uganda National Council for Science and Technology (HS5575ES), for DRC from the Institutional Committee of Health ethics (UCB/CIES/PB/001/2025) and for Tanzania from the NIMR (NIMR/HQ/R.8a/Vol.IX/5187).

Study results will be published in peer-reviewed international journals, and publication will be according to the International Committee of Medical Journal Editors recommendations. Furthermore, results will be presented at national and international conferences. The investigators oblige themselves to publish both positive and negative findings.

## Discussion

Understanding the durability of vaccine-induced immunity in populations affected by the ongoing clade I mpox epidemic remains a critical unresolved question. MpoxVax AFRIVAC was designed to generate longitudinal immunological data in African settings where the current burden of disease is greatest and where host immune context may differ substantially from populations studied previously. Most evidence on MVA-BN immunogenicity derives from studies conducted during the 2022 clade IIb outbreak in high-income regions, with limited representation of African populations, women or immunocompromised individuals.^22^ This evidence gap is particularly relevant given that host genetic diversity, background pathogen exposure and immune status may influence vaccine responses in African settings.^23^ Generating immunological data in affected populations is therefore essential to assess generalisability of prior findings and inform vaccination strategies where mpox burden is highest.

A major strength of this study is its deliberate inclusion of populations under-represented in prior mpox vaccine research despite bearing a substantial burden of disease. Recruitment quotas ensure strong representation of women and PWH, allowing assessment of immune responses in groups at increased risk of infection and severe outcomes.^21 24^ In the ongoing clade I epidemic, women account for a large proportion of cases, and transmission to children may occur through both vertical infection and close caregiving contact, contributing to severe disease in infants and young children.^25 26^ In parallel, accumulating evidence suggests that PWH, particularly those with advanced HIV, may mount attenuated or less durable antibody responses following vaccination.^10 18 19^ Characterising immune responses in these populations is therefore essential to determine whether vaccine-induced protection varies by host immune status and to inform vaccination strategies in settings where both mpox and HIV remain highly prevalent.

The harmonised design with the parallel European MpoxVax study further strengthens the study by enabling comparison of immune kinetics across distinct host and epidemiological contexts. Such comparisons may help clarify whether differences in vaccine responses reflect host immune status, exposure intensity or background immunological context. By generating longitudinal immunogenicity data in populations most affected by the current epidemic, this study aims to inform optimisation of vaccination strategies, including the potential need for targeted booster approaches in vulnerable groups.

This study has several limitations. Implementation of the study has necessarily reflected the operational constraints of the outbreak response. Limited vaccine availability meant that the planned Tanzanian site has been unable to initiate enrolment, and vaccination programmes in Uganda and the DRC proceeded with a single-dose strategy. Although this results in heterogeneity in vaccine exposure, it mirrors the conditions under which mpox vaccination is currently being deployed in endemic settings. Studying immune responses within this context provides an opportunity to characterise the magnitude and durability of responses following a single MVA-BN dose in a clade I transmission setting, data that remain scarce but are highly relevant for policy decisions where vaccine supply is constrained. Furthermore, as an observational cohort embedded within national vaccination programmes, it cannot directly estimate vaccine effectiveness or causal protection. Cellular immune responses, which likely contribute substantially to protection against OPXVs, will not be comprehensively characterised in all participants.

Despite these limitations, the MpoxVax AFRIVAC study will provide urgently needed longitudinal data on MVA-BN vaccine immunogenicity in populations at the centre of the current mpox epidemic. By generating harmonised data across African and European populations, the study aims to clarify how host and epidemiological factors shape vaccine-induced immunity. These insights will be critical for informing vaccination strategies, guiding booster policies and supporting equitable, evidence-based mpox control globally.

## Supplementary material

10.1136/bmjopen-2026-120064 online supplemental file 1
