# Supplementary material for: Examining the immune response of participants receiving Modified Vaccinia Ankara vaccine in Africa (MpoxVax AFRIVAC): a prospective cohort study – protocol
Source: BMJ Open. 2026 Jul 31;16(7):e120064. doi: 10.1136/bmjopen-2026-120064 (PMC13436077; doi:10.1136/bmjopen-2026-120064)
Supplement: Supplementary file 1 [file bmjopen-2026-120064.reviewer_comments.pdf]

PEER REVIEW HISTORY

BMJ Open publishes all reviews undertaken for accepted manuscripts. Reviewers are asked to complete a checklist review form and are provided with free text boxes to elaborate on their assessment. These free text comments are reproduced below.

ARTICLE DETAILS

Title (Provisional)

A protocol for examining the immune response of participants receiving Modified Vaccinia Ankara vaccine in Africa (MpoxVax AFRIVAC): A prospective cohort study

Authors

Byrne, Joanne; Muttamba, Winters; Balola Ntaboba, Alain; Mtafya, Bariki; Bbuye, Mudarshiru; Kalyebi Watelo, Arthur; Alvarez Barco, Elena; Mufudza, Ayleen; Bakamutumaho, Barnabas; Feeney, Eoin R; Obuku, Andrew Ekii; Ntinginya, Nyanda Elias; Katoto, Patrick DMC; Sabiiti, Wilber; Kirenga, Bruce; Mallon, Patrick WG

VERSION 1 - REVIEW

**Reviewer**	**1**
**Name**	**Sharif, Nadim**
**Affiliation**	**Jahangirnagar University, Microbiology**
**Date**	**14-May-2026**
**COI**	**None**

Overall assessment

This manuscript describes a prospective multicentre observational cohort study evaluating durability of immune responses following MVA-BN vaccination in African populations affected by the ongoing clade I mpox outbreak. The study addresses an important global health question, particularly given the limited immunogenicity data available from African settings, women, and people living with HIV (PLWH). The protocol is timely, well written, and operationally relevant.

The longitudinal design, predefined recruitment quotas for underrepresented populations, and harmonisation with the Irish MpoxVax study are notable strengths. The statistical analysis plan is detailed and generally appropriate for the study objectives.

Major comments

Study timeline should be stated more explicitly

The manuscript states that the study is ongoing but does not clearly specify the anticipated recruitment period and follow-up dates. As this is a protocol paper, the planned dates for

recruitment commencement, expected completion of enrollment, and completion of follow-up should be explicitly included.

Clarification regarding vaccine dosing heterogeneity

The protocol notes that some participants may receive only one dose due to vaccine availability, while a second dose may be administered if supplies improve. It would be helpful for the authors to clarify how participants receiving one versus two doses will be handled analytically, and whether subgroup analyses are planned.

Handling of baseline seropositivity

Since prior asymptomatic mpox infection may influence antibody responses, additional clarification is needed regarding:

how baseline seropositivity will be defined,

whether baseline serostatus will be included in longitudinal analyses,

and whether analyses stratified by baseline antibody status are planned.

Cellular immunity analyses

The manuscript mentions that cellular immune analyses may be performed in future studies. It would strengthen the protocol to briefly clarify whether PBMC samples collected in this study are intended for predefined exploratory analyses or solely for future biobanking purposes.

Participant retention

Given the 48-week follow-up period across multiple outbreak settings, a brief description of planned retention strategies would be useful.

Minor comments

Please standardise terminology throughout the manuscript (e.g., "PWH" vs "PLWH").

Several minor grammatical issues should be corrected. Examples include:

"Approval for Uganda have been obtained" → "Approval for Uganda has been obtained."

"community mobilisers boost recruitment" → "community mobilisers to boost recruitment."

Clarify whether HIV-related clinical variables (e.g., CD4 count, viral load, ART status) will be collected for subgroup analyses.

The rationale for selecting week 6 as the change point in the mixed-effects model could be briefly explained.

Figure 1 may benefit from minor formatting improvements to improve readability.

Please clarify whether neutralisation assays will be performed on all participants or only selected subsets.

VERSION 1 - AUTHOR RESPONSE

Reviewer: 1
Comments to the Author:
Overall assessment

This manuscript describes a prospective multicentre observational cohort study evaluating durability of immune responses following MVA-BN vaccination in African populations affected by the ongoing clade I mpox outbreak. The study addresses an important global health question, particularly given the limited immunogenicity data available from African settings, women, and people living with HIV (PLWH). The protocol is timely, well written, and operationally relevant.
The longitudinal design, predefined recruitment quotas for underrepresented populations, and harmonisation with the Irish MpoxVax study are notable strengths. The statistical analysis plan is detailed and generally appropriate for the study objectives.

Major comments

Study timeline should be stated more explicitly
The manuscript states that the study is ongoing but does not clearly specify the anticipated recruitment period and follow-up dates. As this is a protocol paper, the planned dates for recruitment commencement, expected completion of enrollment, and completion of follow-up should be explicitly included.

We have now clarified the study timeline within the manuscript, including the timing of recruitment initiation, site implementation, and anticipated completion of participant follow-up (Page 12). Specifically, recruitment commenced in Uganda and the Democratic Republic of the Congo following regulatory approvals and site initiation activities in January 2025. Planned recruitment in Tanzania did not proceed due to limited vaccine availability, and completion of final participant follow-up is anticipated in June 2026.

Clarification regarding vaccine dosing heterogeneity
The protocol notes that some participants may receive only one dose due to vaccine availability, while a second dose may be administered if supplies improve. It would be helpful for the authors to clarify how participants receiving one versus two doses will be handled analytically, and whether subgroup analyses are planned.

We have clarified within the manuscript that all participants included in this study received a single dose of MVA-BN vaccination as part of national vaccination programmes implemented during a period of constrained vaccine supply (Page 10-11, Figure 1). Accordingly, all primary and secondary immunogenicity analyses described in this protocol relate to single-dose MVA-BN vaccination, and subgroup analyses by vaccine dose number are not planned.

Handling of baseline seropositivity
Since prior asymptomatic mpox infection may influence antibody responses, additional clarification is needed regarding:
how baseline seropositivity will be defined,
whether baseline serostatus will be included in longitudinal analyses,
and whether analyses stratified by baseline antibody status are planned.

Thank you for this important comment. We have now clarified within the Statistical Analysis section (Page 18) how baseline seropositivity will be defined and incorporated into analyses. The proportion of participants with detectable OPXV-specific binding IgG at week 0 consistent with prior mpox infection is explicitly described as a predefined secondary endpoint. Baseline seropositivity will be defined using pre-specified MPXV B6R IgG seropositivity thresholds derived from our previously validated electrochemiluminescence immunoassay. We have additionally clarified that, as these thresholds were originally derived in non-African populations, their performance and applicability within the African cohort will be evaluated and the seropositivity threshold may be revised if required based on observed assay performance characteristics in this setting. We have additionally clarified that baseline serostatus may be incorporated as an exploratory covariate in analyses evaluating factors associated with vaccine-induced immune responses.

Cellular immunity analyses
The manuscript mentions that cellular immune analyses may be performed in future studies. It would strengthen the protocol to briefly clarify whether PBMC samples collected in this study are intended for predefined exploratory analyses or solely for future biobanking purposes.

We have now clarified within the manuscript that PBMC samples are being prospectively collected to support exploratory cellular immunological analyses in selected participant subsets, and for future ethically approved ancillary studies related to mpox immunology and vaccine responses (Page 13).

Participant retention
Given the 48-week follow-up period across multiple outbreak settings, a brief description of planned retention strategies would be useful.

We thank the reviewer for this suggestion. We have now included a brief description of participant retention strategies within the manuscript. These included ongoing community engagement and sensitisation activities led by local study teams, RCCE teams, and Community Advisory Boards (CABs), alongside flexible visit scheduling and regular participant communication throughout follow-up to support long-term participant retention across study sites (Page 12).

Minor comments
Please standardise terminology throughout the manuscript (e.g., “PWH” vs “PLWH”).

This has been standardised throughout the manuscript to People with HIV (PWH).

Several minor grammatical issues should be corrected. Examples include:
“Approval for Uganda have been obtained” → “Approval for Uganda has been obtained.”

This has been updated (Page 20).

“community mobilisers boost recruitment” → “community mobilisers to boost recruitment.”

This has been updated (Page 9).

Clarify whether HIV-related clinical variables (e.g., CD4 count, viral load, ART status) will be collected for subgroup analyses.

Thank you for this important comment. We have clarified the HIV-related clinical variables collected within the study. ART status, concomitant medications, and history of advanced HIV disease (defined as HIV infection with CD4+ T-cell count <200 cells/mm^3) will be collected for participants with HIV (Page 11). We have additionally clarified that these variables may be evaluated as exploratory covariates in analyses assessing factors associated with vaccine-induced immune responses (Page 18).

The rationale for selecting week 6 as the change point in the mixed-effects model could be briefly explained.

We have now clarified the rationale for selecting Week 6 as the pre-specified change point within the mixed-effects model (Page 16). Specifically, we now state that Week 6 was selected a priori as it is expected to approximate peak vaccine-induced antibody responses following MVA-BN vaccination, thereby separating the early antibody expansion phase from the subsequent waning phase.

Figure 1 may benefit from minor formatting improvements to improve readability.
Figure 1 has been reformatted to improve readability and clarity. Revisions include improved table layout and spacing, simplified visit labelling, and clearer presentation of the study visit schedule and sampling procedures. We have also clarified that one dose of MVA-BN vaccine is administered.

Please clarify whether neutralisation assays will be performed on all participants or only selected subsets.

This has been clarified that functional neutralisation assays may be performed on selected subsets of stored samples in future studies (Page 13).
