# Supplementary material for: Examining the immune response of participants receiving Modified Vaccinia Ankara vaccine in Africa (MpoxVax AFRIVAC): a prospective cohort study – protocol
Source: BMJ Open. 2026 Jul 31;16(7):e120064. doi: 10.1136/bmjopen-2026-120064 (PMC13436077; doi:10.1136/bmjopen-2026-120064)
Supplement: Supplementary file 2 [file bmjopen-16-7-s001.docx]

**Supplementary Figure 1: Schedule of visits for participants who have received first dose of MVA-BN <28 days previously**

Subjects will attend a total of 4 clinical trial visits over 48 weeks of study as outlined in the schedule of visits above. Visit 1 should be scheduled within a window period of ±3 days, visit 2 within a window period of ±7 days, while visit 3 and visit 4 should be scheduled within a window period of ±2 weeks. Week of visit is calculated post date of first MVA dose.

*Unscheduled visit to facilitate diagnosis and evaluation of mpox (vaccine failure cases) or evaluation of adverse events. Unscheduled visits will include clinically indicated laboratory testing and if mpox is suspected, biobanking of plasma, serum and PBMCs and swab of mpox lesions for future related work
